# Tinnitus, Sudden Sensorineural Hearing Loss, and Vestibular Neuritis As Complications of the Astra Zeneca COVID-19 Vaccine

**DOI:** 10.7759/cureus.20906

**Published:** 2022-01-03

**Authors:** Mario Canales Medina, Mariana Ramirez Gómez

**Affiliations:** 1 Otolaryngology - Head and Neck Surgery, Centro Medico Dalinde, Mexico City, MEX; 2 Medicine, Centro Medico Dalinde, México City, MEX

**Keywords:** covid-19 vaccines, astra zeneca covid vaccine, vestibular neuritis, tinnitus, sensorineural hearing loss

## Abstract

Background: Sudden sensorineural hearing loss is most commonly defined as a sensorineural hearing loss of 30dB or greater over at least three contiguous audiometric frequencies occurring within a 72-hr period. The Astra Zeneca COVID-19 vaccine is suspicious of causing thrombotic complications following its administration, and could theoretically induce hearing loss by damaging the hearing organs through this mechanism, as well as vestibular damage through similar mechanisms.

Material and Methods: We reviewed the files of patients with otological symptoms after exposure to the Astra Zeneca COVID-19 vaccine during the year 2021.

Case Series: We studied a total of six cases with otologic symptoms temporally related to the Astra Zeneca COVID-19 vaccine. We report four cases of patients presenting with hearing loss and tinnitus a few days after the second dose of the Astra Zeneca vaccine, and one case with the same symptoms after the first dose. Four cases were successfully treated with steroids; however, one case presented to the office two months after the onset of symptoms and did not improve with treatment. We also report the first case of vestibular neuritis temporally related to the administration of the first dose of the vaccine, which also had a good outcome after medical treatment.

Conclusions: Prompt treatment in the present cases was a factor associated with a good prognosis.

## Introduction

Sudden sensorineural hearing loss (SSHL) is a disease characterized by sensorineural hearing loss (SHL) of unknown cause, most commonly defined as an SHL of 30dB or greater over at least three contiguous audiometric frequencies occurring within a 72-hr period [1,2]. Although its pathogenesis remains unclear, a plausible hypothesis (among many) is ensuing reduced perfusion to the hearing organs due to ischemia, which could be the case in patients receiving the Astra Zeneca COVID-19 vaccine (AZCV), which is suspicious of causing thrombotic complications following its administration; regardless of the cause of SSHL, systemic administration of glucocorticoid steroids and intratympanic therapy with steroids (ITS) are accepted treatments [2-4]. ITS is considered more effective with respect to the drug delivery system, especially in the context of the COVID-19 pandemia [2,5].

Several previous reports have mentioned the possible link between COVID-19 infection and inner ear damage [2,6-10]; however to the authors´ very best knowledge this is the first case series considering the possible association between AZCV (or any COVID-19 vaccine) and inner ear damage, and so far only two previous isolated single case reports of auditory symptoms related to the AZCV have been described, including one report of SSHL itself [11], and one report of tinnitus presumably associated to cochleopathy secondary to the vaccine [12]. To our best knowledge, this is the first report of a patient with vestibular symptoms related to the AZCV.

## Materials and methods

Study design: This is a retrospective transversal observational study described as case series.

Data collection: The clinical files of patients presenting to our practice in 2021 (the year that the COVID-19 vaccination program started in our country) with auditory and/or vestibular symptoms after the administration of the AZCV were searched in our database and reviewed, obtaining a total of six files for revision. 

Data analysis: Patients were grouped according to their main otologic symptoms as auditory or vestibular, yielding a total of five patients with auditory symptoms and one patient with vestibular symptoms. No further statistical tests were possible given the small number of cases that we had. We describe cases in detail one by one.

## Results

Case series

**Figure 1:**
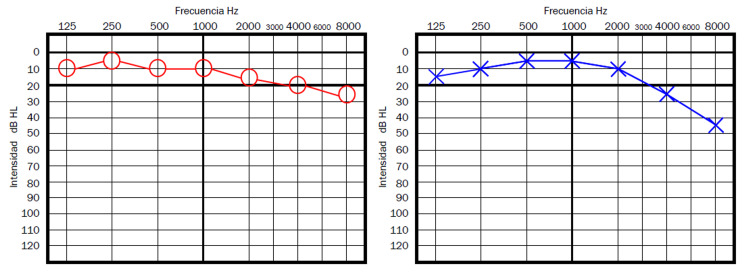
Patient 1 - Pure tone audiometry showing an evident hearing loss in the left ear in high tones compared to the contralateral ear

The first case is a 61-year-old male patient who presented to the Otolaryngology Clinic in July 2021, referring SSHN associated with severe high pitched tinnitus in the left ear for one month before that initial visit; he had the second dose of the AZCV 10 days before the onset of the otologic symptoms. Pure tone audiometry (PTA) performed 10 days after the onset of symptoms revealed a moderate SHL in the left ear in high frequencies (Figure 1). Although the traditionally accepted criteria for SSHL were not met, it was evident that he had a descent in the hearing thresholds in high frequencies comparing the left ear (affected) with the right ear (asymptomatic). He had no specific treatment with steroids for management of the hearing loss and tinnitus before that first visit; after discussing the options with the patient, it was decided to use a tympanostomy tube on the left ear as definitive urgent management for ITS with dexamethasone; within the first three days after ITS, he reported significant improvement both in hearing and in tinnitus perception, being now resolved the tinnitus, and having almost normal control audiometry.

**Figure 2:**
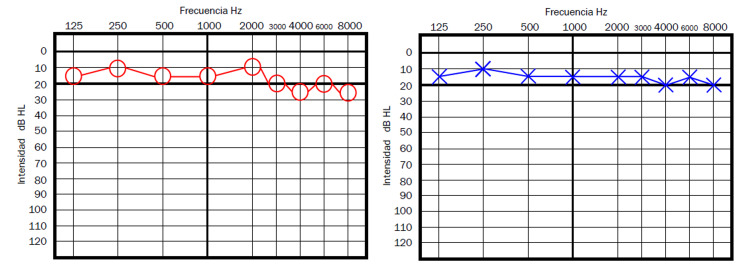
Patient 2 - Pure tone audiometry showing a superficial hearing loss in the right ear (the ear affected with high pitched tinnitus), with a loss of approximately 5dB in high frequencies compared to the left ear

The second case is a 45-year-old female, who presented to the Otolaryngology Clinic just a few days after the first patient. She had the same symptoms as the first patient for five days before our initial meeting. In this second case, the right ear was affected. She also had the second dose of the AZCV 10 days before the onset of symptoms. On PTA performed that same day of that first visit, she had a superficial SHL in the right ear just 5dB less than the other ear on 3, 4, 6, and 8kHz (Figure 2); therefore, the traditional criteria for SSHL were not met either. After discussing the options, considering that she had less time since the onset of symptoms, less severe hearing loss, and less severe tinnitus, it was decided to manage the case with oral prednisone (1mg/kg/day) initially. She reported the resolution of symptoms within the first three days also.

**Figure 3:**
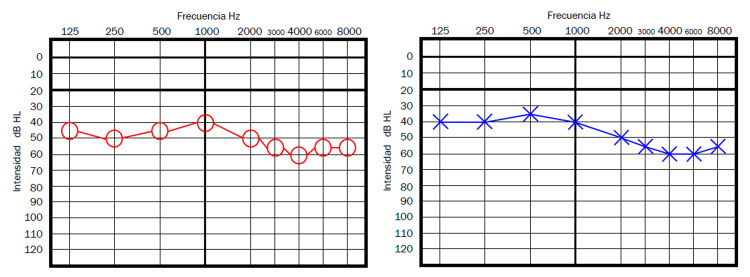
Patient 3 - Initial pure tone audiometry

**Figure 4:**
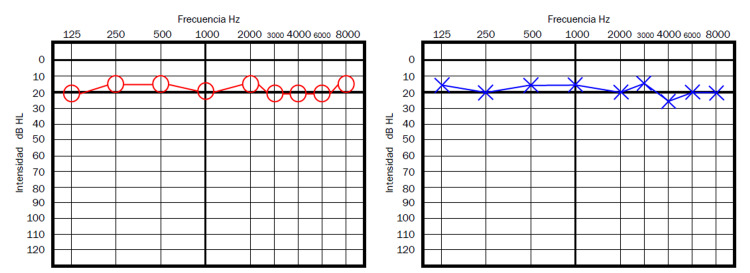
Patient 3 - Pure tone audiometry after three weeks of oral prednisone

Case 3 is a 44-year-old male patient, who presented to our clinic 12 days after the onset of bilateral hearing loss and tinnitus; those symptoms started approximately 18 days after the second dose of the AZCV. A moderate bilateral hearing loss (average hearing threshold of 50dB on both ears (Figure 3), therefore consistent with the traditional SSNHL criteria. He was managed with oral prednisone 1mg/kg/day single dose and after three weeks, tinnitus had disappeared and he showed a normal audiometry study by then (Figure 4).

**Figure 5:**
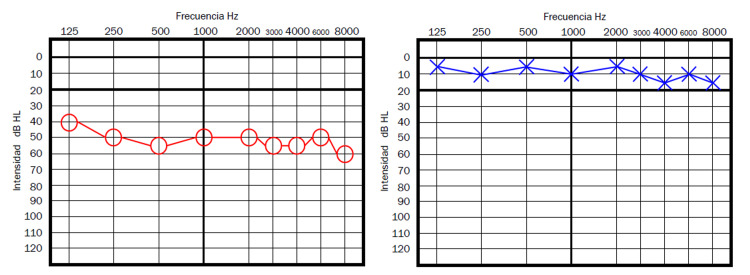
Patient 4 - Pure tone audiometry showing a moderate hearing loss on the right ear, consistent with the traditional criteria for SSNHL SSNHL - sudden sensorineural hearing loss

Case 4 is a 39-year-old male patient who reported both tinnitus and sudden hearing loss on the right ear, 11 days after the first dose of the AZCV. On audiometry, he had normal hearing on the left ear, and a moderate right hearing loss (average hearing threshold of 50dB on right ear), consistent with the traditional SSNHL criteria (Figure 5). He was managed with oral prednisone 1mg/kg/day single dose and after three weeks, tinnitus had disappeared and he showed a normal audiometry study by then.

**Figure 6:**
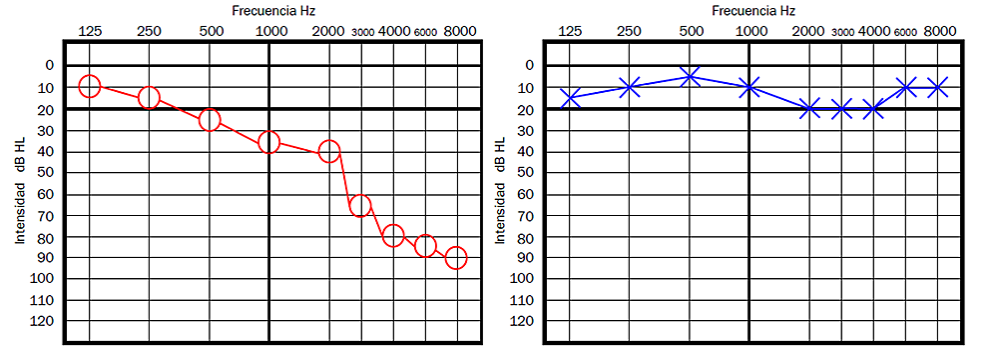
Patient 5 - Pure tone audiometry showing a sensorineural hearing loss in the right ear, consistent with the classical criteria for SSNHL SSNHL - sudden sensorineural hearing loss

Case 5 is a 43-year-old male patient who started two months before coming to the clinic with right ear tinnitus and hearing loss, he had received the second dose of AZCV approximately 14 days prior to the onset of symptoms. On audiometry, he had normal hearing on the left ear and a severe hearing loss on the right ear, more severe in high frequencies reaching 90dB of hearing threshold on 8,000Hz (Figure 6). He was managed with oral prednisone 1mg/kg/day single dose and after one week, he reported no improvement neither in the hearing loss nor in tinnitus and after discussing the options with the patient, treatment was stopped; the patient was lost to follow up and no control audiometry could be obtained.

Case 6 is a 40-year-old woman presented to the emergency department of our institution, referring to having received the first dose of AZCV three weeks before. She had at that time severe vertigo associated with nausea, vomiting, and impossibility to walk. Upon examination, she had grade III horizontal nystagmus with rapid phase toward the left side showing all the classic features described in vestibular neuritis (VN), right lateropulsion, and ocular tilt reaction, therefore, presenting the classic clinical signs and symptoms described in VN [13] and was diagnosed as a right VN. Due to the severity of symptoms and to our institution´s treatment protocols, no vestibular testing could be obtained at that time. Considering the severity of the symptoms and the impaired oral intake she was admitted for medical treatment, being managed with intravenous diphenidol, ondansetron, and dexamethasone, and discharged home after 48hr. After one week (nine days after the onset of symptoms), she presented to the office for follow-up with only slight instability; by then nystagmus and lateropulsion (even during Fukuda test) had disappeared. After one month of follow-up, she developed benign paroxysmal positional vertigo on the same side (right posterior canal) and was successfully managed with the Epley maneuver.

## Discussion

This is the first case series reporting damage to the hearing organs probably related to a vaccine for COVID-19. Although more evidence is yet to be found, the temporal relation between the AZCV exposure and the appearance of hearing loss and tinnitus a few days after the vaccination in all cases clearly points toward a likely association between the vaccine and the otologic symptoms. Many authors have considered a possible link between COVID-19 infection and SSHL [2,6-10]; however, as far as the author knows this is the first case series considering the possible association between a COVID-19 vaccine and inner ear damage, which could indeed have pathophysiology similar to that of the cases of SSHN secondary to COVID-19 infection. We display the audiometries of cases 1 and 5 since we believe them to be the most representative of the situation described by Tseng et al. [12] (case 1) and the one described by Tsetsos et al. [11] (case 5). Even if two cases presented here did not clearly show the traditional full criteria for SSHL, the presentation was very similar to a traditional SSHL and both were treated as such by using steroids similar to what was described by Tseng et al. [12], with most cases having the resolution of the symptoms around the day 3 of management. The auditory symptoms were more commonly related to the second dose of the AZCV (four cases had the onset of symptoms after the second dose and one after the first dose) and started on average 11 days after the administration of the AZCV. The patient with VN managed with dexamethasone, ondansetron and diphenidol also had a relatively good outcome, and it seems reasonable to follow that strategy until further information is available since this is the report of a patient with vestibular symptoms probably related to the AZCV; interestingly the time since exposure to the AZCV to the onset of vestibular symptoms was longer than that for the patients with auditory symptoms. The inner ear damage related to the AZCV can have a very heterogeneous presentation as is described in detail in these cases, and a high index of suspicion is mandatory, in order to start immediate treatment to improve the prognosis. Auditory damage seems to be more common than damage to the vestibular organs after exposure to the AZCV. Unfortunately, we could not get vestibular testing in the acute phase for case 6, which would have been interesting for research purposes, nevertheless, we had strong clinical evidence for the diagnosis of VN, which was temporally related to the AZCV, and symptoms improved with medical treatment. The AZCV has many possible side effects, even if the pathophysiology remains obscure, here we clearly observed inner ear damage as a probable side effect directly related to the AZCV. Further studies will be needed to confirm this hypothesis and to better understand the underlying pathophysiology. Due to the small size of our case series, for now, it is only possible to make a descriptive analysis with the available information to this date.

## Conclusions

We present six cases of patients with otologic symptoms probably related to the AZCV, all of them with the onset of symptoms a few days after the AZCV exposure; five confirmed to have hearing loss by PTA, and one with clinical criteria strongly consistent with acute VN.

A high index of suspicion must be kept in mind for inner ear damage in patients with otologic symptoms after the administration of the AZCV; we recommend these group of patients get a PTA as soon as possible in case of audiological symptoms and start steroid therapy immediately to prevent further damage to the inner ear even if the strict criteria for SSHL are not fully met, in the case of VN it seems reasonable to start treatment as with any other acute VN, at least until further studies are available.

Hearing loss and tinnitus can be solved in this context by using appropriate steroid therapy as would be done with any other SSHL. Delaying the treatment with steroids seems to be a bad prognostic factor for recovery in this group of patients with auditory symptoms. VN in this context also seems to have a relatively good prognosis. Pathophysiology of inner ear damage associated with AZCV is yet to be established, further studies are needed to confirm the association of hearing loss, tinnitus, and VN with the AZCV, and to define the optimal treatment protocols in these cases.

Damage to the inner ear must be considered as a possible side effect of the AZCV, a high index of suspicion must be observed in this context, and treatment should be started accordingly as soon as possible.
